# Mental Health Component Scale Is Positively Associated with Riboflavin Intake in People with Central Obesity

**DOI:** 10.3390/nu15204464

**Published:** 2023-10-21

**Authors:** Charalampia Amerikanou, Aristea Gioxari, Stamatia-Angeliki Kleftaki, Evdokia Valsamidou, Antonia Zeaki, Andriana C. Kaliora

**Affiliations:** 1 Department of Nutrition and Dietetics, School of Health Science and Education, Harokopio University of Athens, 70 El. Venizelou Ave., 17676 Athens, Greece; amerikanou@windowslive.com (C.A.); matina.kleftaki@gmail.com (S.-A.K.); evdokiavalsamidou@gmail.com (E.V.); antonia_zeaki@hotmail.gr (A.Z.); 2 Department of Nutritional Science and Dietetics, School of Health Sciences, University of the Peloponnese, Antikalamos, 24100 Kalamata, Greece; a.gioxari@uop.gr

**Keywords:** mental health, obesity, micronutrients, vitamins, minerals, riboflavin

## Abstract

Micronutrient deficiencies are a well-established fact in obesity. However, few studies exist on the relationship between micronutrient intake and mental health. In this study, we investigated the associations between daily intakes of vitamins and minerals and scoring items that measure mental health in people living with central obesity. One hundred males and females with central obesity and metabolic abnormalities were included in the study. Demographic, clinical, anthropometric, and biochemical data were collected. Mental health statuses were assessed with validated questionnaires, and daily micronutrient intakes were assessed with food diaries and Nutritionist Pro^TM^ software v7.9. The mental component score (MCS-12) positively correlated with vitamin A (Rho = 0.249, *p* = 0.038), vitamin C (Rho = 0.293, *p* = 0.014), riboflavin (Rho = 0.264, *p* = 0.026), and folate (Rho = 0.238, *p* = 0.046). Rosenberg Self-Esteem Scale (RSES) correlated with sodium (Rho = 0.269, *p* = 0.026), and the Center for Epidemiologic Studies Depression Scale Revised (CESD-R) correlated with chromium (Rho = 0.313, *p* = 0.009). In the regression analysis, after potential confounders were adjusted for, only riboflavin was positively associated with the MCS-12 log (beta ± SD = 0.047 ± 0.023, *p* = 0.044). Our study provides evidence of the link between dietary riboflavin and mental health in people with obesity, and it highlights the importance of monitoring both nutritional status and mental health when managing obesity.

## 1. Introduction

Over the past 50 years, there has been a noticeable rise in obesity, which is defined as an excessive accumulation of fat. Obesity has emerged as a “global pandemic”, with more lives claimed by obese people compared to underweight people [1,2]. Type 2 diabetes mellitus, metabolic syndrome, nonalcoholic fatty liver disease (NAFLD), and various types of cancer are all strongly correlated with obesity [1] and place a significant burden on the socio-economic system both directly and indirectly [1]. Obesity, which is also regarded as a chronic inflammatory disorder with a compromised immune system, has many drivers including unhealthy eating habits and a sedentary lifestyle in combination with genetic, epigenetic, metabolic, and environmental factors [3].

Obesity is characterized by a paradox. Despite their excessive energy consumption, people living with obesity are usually malnourished due to their insufficient intake or reduced absorption of essential nutrients [4]. A reason for their insufficient supply of essential nutrients (despite overfeeding) is the fact that modern agricultural systems focus on intensive food production and quantity rather than the quality of the produce, which leads to the production of nutrient-deficient foods [5]. Furthermore, micronutrient deficiencies can also be caused by deviations in nutrient absorption and micronutrient metabolism, a result of the systemic inflammation associated with obesity [4].

Several micronutrient deficiencies have been described in people living with obesity, including minerals such as calcium, iron, zinc, and selenium, and antioxidant vitamins such as C and E, or the vitamin B complex. The metabolic and regulatory pathways, such as energy metabolism and protein synthesis, require the presence of cofactors and vitamins; therefore, their deficiencies may impair human health. For example, B vitamins are considered regulators of several immune functions, and their deficiencies have been linked to various cancers [6]. In addition, they are known as“neurotropic” vitamins since they exhibit neurospecific functions and have a significant role in both the central and peripheral nervous systems. It is widely recognized that diet, through the supply of nutrients, has a significant effect on the normal functioning of the central and peripheralnervous systems. Several neurological diseases, such as depression and peripheral neuropathy, have been linked with B vitamin deficiencies [7]. Moreover, diabetes may develop in obese people due to deficiencies in chromium, vitamin C, and vitamin D, which are necessary for insulin signaling pathways and glucose metabolism, respectively [8]. In addition, zinc is an essential element for the metabolism of foods, protein synthesis, and body mass conservation. Its levels are often lower in obese people, which indicate a loss of lean body mass [9]. Furthermore, selenium regulates multiple processes that are usually impaired in obesity. These include thyroid and immune functions, carbohydrate and lipid metabolism, and more importantly, adipose tissue molecular pathways involving adipogenesis, lipolysis, oxidative stress, and inflammation [10].

Thus far, few studies have addressed the relationship between mental health and micronutrient intake in people with obesity and metabolic abnormalities. In a previous study, we demonstrated that mental health indices are related to markers of inflammation in central obesity [11]. Thus, considering that low-grade chronic inflammation and micronutrient deficiencies are evident in obesity and that micronutrients are implicated in the regulation of immune function and inflammatory pathways, we aimed to evaluate the association between dietary micronutrient intake (vitamins and minerals) and mental health in Greek adults with central obesity and related metabolic disorders.

## 2. Materials and Methods

### 2.1. Study Design and Participants

This cross-sectional study included data from metabolically unhealthy obese subjects that participated in a trial assigned the identifier NCT04785573 to clinicaltrials.gov. Specifically, the cohort consisted of 100 people, male and female, with cardiometabolic parameters associated with the metabolic syndrome and a complete nutritional record. Moreover, the adults recruited had been diagnosed with central obesity, which is defined as a waist circumference (WC) of>94 cm in males and >80 cm in females, concurrent with a minimum of one metabolic disorder related to obesity, including a triglycerides (TG) level of ≥150 mg/dL, or a high-density lipoprotein (HDL) cholesterol measurement ≤40 mg/dL in the men and ≤50 mg/dL in the women, or an increased blood pressure of ≥130/85 mm Hg, or an elevated fasting blood sugar level ≥100 mg/dL. The exclusion criteria included any type of surgery for weight loss or restrictive diet for weight loss in the 3 months prior to enrollment, pregnancy, lactation, untreated thyroid disease, the use of supplements in the 3 months prior to the recruitment, drug and/or alcohol abuse, and the diagnosis of any psychiatric/mental disorders. In addition, subjects were required to have had a stable body weight for a minimum of 3 months prior to the study and to have a moderately active lifestyle.

The study protocol adhered to the principles set out in the updated revisions of the Declaration of Helsinki and the Data Protection Act, and it acquired the necessary ethical approval from the Harokopio University Ethics Committee (ID protocol: 1799/13 June 2019). All participants were well-informed about the study and were asked to sign a non-binding letter of consent.

### 2.2. Medical, Demographic, Anthropometric, Lifestyle, and Biochemical Assessment

A thorough medical history was taken along with demographics (age, sex, and marital status) and lifestyle information (diet, smoking, and physical activity). Furthermore, anthropometric indices were measured (weight, height, and waist circumference), and by dividing the weight (in kg) by the square of the height in meters (m^2^), the body mass index (BMI) was calculated. For the evaluation of physical activity, the International Physical Activity Questionnaire Short Form (IPAQ-SF) was used.The results were presented as the metabolic equivalent task minutes per week (MET-min/week) [12].

Qualified dietitians assessed the dietary habits of all subjects. We utilized a 24-h dietary recall to obtain quantitative data on the micronutrient intake over the course of four days, two working days, and one weekend. The analyses and quantification were performed using Nutritionist Pro^TM^ software v7.9 (Axxya Systems Nutritionist Pro™, Stafford, TX, USA).

After overnight fasting, 20 mL of blood was drawn from each individual. Vacutainers were centrifuged at 3000 rpm for 10 min. The supernatant serum was stored at −80 °C, and as required, the serum was thawed and used for biochemical measurements of glucose, lipids, and hepatic function with an automatic biochemical analyzer (Cobas 8000 analyzer, Roche Diagnostics GmbH, Mannheim, Germany).

### 2.3. Mental and Physical Health Assessment

The validated questionnaires were used to assess general qualityoflife characteristics. The 12-item Short Form Survey (SF-12) isself-reported.It contains two scores, one for physical health (Physical Component Score, PCS-12) and the other for mental health (Mental Component Score, MCS-12) [13]. The Center for Epidemiologic Studies Depression Scale Revised (CESD-R) was used to examine the prevalence of depression in our cohort [14]. This self-reported questionnaire includes 20 items covering mood, somatic complaints, social interactions, and motor performance. Each item has a scoring range of 0 to 3, and overall scores can reach a total of between 0 and 60; scores of 16 or more indicate a risk of severe depression. Self-esteem was assessed by the most widely used measure of global self-esteem, the 10-item Revised Rosenberg Self-Esteem Scale (RSES). The final score ranges from 0 to 40, with scores for each item varying from strongly agree to strongly disagree. A total score below 15 indicates low self-esteem [15].

### 2.4. Statistical Analysis

In this study, the normally distributed quantitative variables are presented as mean ± standard deviation (SD), whereas those that are not normally distributed are presented as median variables (interquartile range, IQR). The qualitative variables are presented as counts (%). Furthermore, the correlation analysis of the mental and physical health parameters with the dietary intake of micronutrients was performed with either a Pearson correlation test or a Spearman’s rank correlation test, depending on the distribution. Additionally, we applied multivariate linear regression models to test the associations with significant bivariate correlations, adjusting for possible confounders and logarithmically transforming the variables where needed. The statistical analysis was performed using SPSS 21.0 (IBM, SPSS Inc., Chicago, IL, USA), and *p*-values ≤ 0.05 were considered significant.

## 3. Results

The basic characteristics of the study population are presented in Table 1. The average age was 54.2 ± 11.9 years with a BMI score of 31.6 (8.5) kg/m^2^. The majority was female (61.0%), nonsmokers (83.0%), and married (76.0%). Most participants had hyperlipidemia (88.0%).

Table 2 presents the micronutrient intakes assessed using food diaries and Nutritionist Pro software v7.9 (Axxya Systems Nutritionist Pro™, Stafford, TX, USA, distributed to Harokopio University of Athens). Supplementary Table S1 contains the micronutrient intakes in males and females along with their respective Recommended Dietary Allowances (RDAs) and Adequate Intakes (AIs) [16,17]. In total, the intake of vitamins A, C, D, E, K, cobalamin, folate, biotin, pantothenic acid, and choline, in addition to those of calcium, potassium, magnesium, copper, manganese, phosphorus, zinc, and molybdenum, was lower than the recommended intakes in both sexes. Pyridoxine, riboflavin, and thiamin were present in inadequate quantities in males, while niacin was present in inadequate quantities in females. Moreover, RDAs do not exist for beta-carotene, alpha-carotene, lutein (+zeaxanthin), beta-cryptoxanthin (µg), and lycopene.

The results of the correlation analysis between the dietary intake of micronutrients and the mental and physical health parameters are presented in Table 3. More specifically, MCS-12 positively correlated with vitamin A (Rho = 0.249, *p* = 0.038), vitamin C (Rho = 0.293, *p* = 0.014), riboflavin (Rho = 0.264, *p* = 0.026), and folate (Rho = 0.238, *p* = 0.046). In addition, RSES correlated with sodium (Rho = 0.269, *p* = 0.026) and CESD-R with chromium (Rho = 0.313, *p* = 0.009).

Furthermore, we used three different linear regression models to analyze the significant associations found in the correlation analysis. The first was unadjusted, the second was adjusted for age, sex and BMI, and the final model was adjusted for age, sex, BMI, physical activity level, smoking and medication (antihypertensive, antidiabetic, or statins). It is notable that, even after the final model adjustment, riboflavin was positively associated with the MCS-12 log, as seen in Table 4 (beta ± SD = 0.047 ± 0.023, *p* = 0.044). Moreover, applying regression models and controlling for the relevant confounders revealed no significant relationships in any of the other notable correlations.

## 4. Discussion

Recent studies suggest that mental disorders and obesity have a bidirectional relationship and that they may share some common genetic variants. Bariatric patients exhibit an increased prevalence of depression (19%) and anxiety disorders (12%), whereas the prevalence of obesity ranges from 20% to 50% in depression [18]. In addition, poor diet quality and the subsequent nutritional deficiencies—owed also to altered metabolism, absorption, or pharmacokinetics—contribute to chronic conditions such as type 2 diabetes mellitus and metabolic syndrome [19]. Furthermore, although in the past few decades it has become increasingly clear that obesity is a multifactorial disease and that both metabolic and neurobehavioral features can be affected, this is the first study to look into the relationship between micronutrient intakes and mental health in people with central obesity.

A hundred Greek people with central obesity were examined to create a detailed profile of micronutrients, namely vitamins andminerals. At least one metabolic disorder was present in our sample population, with dyslipidemia being the most common. The daily nutritional intakes were under the RDAs in most of the micronutrients examined in both sexes, corroborating the literature containing supporting evidence of micronutrient deficiencies in people living with obesity.

The most important finding of our study was the positive association between MCS-12 and riboflavin, which remained significant even after adjusting for possible confounders. Riboflavin, also known as vitamin B2, possesses neuroprotective potential. Moreover, it is a neuroactive molecule with immunomodulatory effects, and when it is lacking, brain function is affected; furthermore, its derivatives work as cofactors in the metabolism of the important fatty acids in brain lipids [20,21]. A recent meta-analysis, which included 18 studies on the association of depression with vitamin B complex dietary intake, showed an inverse association between B2 and the risk of depression, which was significant in females but not in males [22]. Although there are many studies on the association between B2 intake and the risk of depression or impaired mental health in the general population, none explore anassociation with scores that reflect mental health in obese adults. Rouhani and his colleagues [23] showed that adults with higher riboflavin intake had decreased odds of having depression and anxiety (as assessed by the Hospital Anxiety and Depression Scale) and psychological distress (as assessed by the General Health Questionnaire (GHQ)). Similarly, in a cross-sectional analysis from the Japan Multi-Institutional Collaborative Cohort Study, dietary vitamin B2 was inversely correlated with the GHQ score in participants with a GHQ score of ≥4 (to which scores of ≥4 reflect poor mental health) [24]. We report for the first time that MCS-12, a validated and reliable measure of mental health for the general population [25], is associated with higher B2 intake in people with central obesity and related metabolic disorders.

The role of the vitamin B complex in obesity and metabolic health is pivotal, as its constituents act as coenzymes in several enzymatic processes and are involved in catabolic energy production and anabolic pathways. B2 is critical for the synthesis, conversion, and recycling of other vitamins and proteins involved in oxygen transport. Its derivatives possess antioxidant properties and are cofactors in the metabolism of fatty acids in brain lipids [26]. It has been shown that riboflavin can inhibit inflammation in adipocyte and macrophage co-cultures, suggesting that its supplementation may regulate inflammation in obesity [27]. In the present population with obesity, we previously showed that obesity-related systemic inflammation is associated with mental health components [11]. Therefore, an increase in micronutrient intake can be associated with antioxidant and anti-inflammatory mechanisms, affecting mental health parameters indirectly. Nevertheless, any lifestyle intervention targeting the nutritional status and metabolic health of people living with obesity offers substantial improvement in their quality of life as well, including mental health [28,29].

The correlation analysis revealed additional relationships of interest between micronutrient intake and the mental health indices. Sodium is positively correlated with RSES, the most common measure for the evaluation of self-esteem. Sodium is an essential micronutrient that is critical for cellular homeostasis and the regulation of blood pressure [30]. Although its role in cardiovascular diseases is well documented, its contribution to mental health is not fully explored. Consuming foods high in sodium has been associated with increased depressive symptoms in adolescents [31], and mice with higher salt intake experienced neuroinflammation and decreased behavioral control [32]. Conversely, hyponatremia, the decrease in serum sodium levels, has been associated with depression symptoms and cognitive impairments [33], suggesting that both excess and deficiency can be detrimental for mental health. Nevertheless, it has been reported that vasopressin not only controls sodium serum concentrations but also acts as a neuromodulator, affecting learning, memory, circadian rhythm, and social behavior [34]. Consequently, sodium intake may be related to cognitive characteristics, such as self-esteem, although in our population the mean sodium intake was within normal levels and this link was not confirmed when adjusting for potential confounders.

Since chromium is recognized for its serotonergic and dopaminergic activity and dietary chromium supplementation improves mood dysregulation in individuals with depression [35], the link between dietary chromium and CESD-R, a measurement of depressive symptoms, was unexpected. However, the relationship between chromium and depression did not survive the regression models to which several adjustments were performed. Similarly, the observed correlations of MCS-12 with vitamin A, vitamin C, and folate were not maintained when linear regression models were applied. Folate is metabolized into L-methylfolate, which crosses the blood–brain barrier and regulates the production of neurotransmitters such as dopamine and serotonin. Moreover, folate deficiency leads to neurological symptoms in adults and neural tube defects in fetuses [36,37,38]. Similarly, vitamin C modulates neurotransmitter synthesis and release, acting as a cofactor in the conversion of dopamine to noradrenaline, regulating dopaminergic neurotransmission, and catecholamine and acetylcholine release [39]. With respect to vitamin A, although it is essential to brain function, caution must be taken in its supplementation as the central nervous system is a clear target of vitamin A-associated toxicity [40].

Anxiety and depression symptoms are prevalent throughout menopause. There have been suggestions that the brain may be affected by variations in hormone levels during the menopausal transition through the effects it has on the hippocampus and hypothalamic function [41]. Depression, irritability, and anxiety have been linked to changes in neuronal opioids after menopause and in serotonin and γ-aminobutyric acid transmission, both of which are influenced by steroid hormones. However, social and psychological risk factors, in addition to changes in steroid hormone levels during the menopausal transition, can cause anxiety and despair [41]. Vasomotor symptoms, neuroticism, stressful life events, a history of major depressive disorders, a low financial or educational level, a lack of social support, and even marital status are a few examples [41]. Conversely, protective factors such as hormone therapy, exercise, and counseling have been proposed. Due to the small sample size, a sub-analysis according to gender, in which the menopause status could be included as a confounder for females, could not be implemented. This should be included in the study’s limitations. Additionally, we acknowledge that a causal relationship between riboflavin intake and mental health could not be established due to the cross-sectional design of the study, in addition to the relatively small sample size of the population used. However, the current study has several strengths, such as the use of validated questionnaires for assessment of mental health, the fact that dietary assessment was performed by experienced dietitians, and the evaluation of several potential confounders in the analysis.

## 5. Conclusions

Herein, we present some interesting, conclusive correlations between the dietary intake of micronutrients and mental and physical health parameters. When adjusting for potential confounders, a positive association emerged between dietary riboflavin and mental health in people living with obesity. Taking into consideration not only the neuroprotective potential of riboflavin but also its antioxidant and anti-inflammatory properties in obesity, our results are of great importance. Although numerous lifestyle interventions have the potential to improve mental health, they lack the reliable biomarkers and mechanistic insights of the above regulations [42]. This highlights further the importance of our findings, as MCS-12 is considered a valid measure of mental health and may be a promising factor when addressing the relationship between nutritional status and mental health in people with obesity and metabolic abnormalities. Notwithstanding the need for further studies with larger samples and the possibility of a prospective or intervention design to establish causality, our study supports the importance of monitoring both nutritional status and mental health when managing obesity.

## Figures and Tables

**Table 1:** Baseline characteristics of the study population.

Variables	N = 100
Age (years), mean ± SD	54.2 ± 11.9
Sex (male/female), N (%)	39 (39.0)/61 (61.0)
BMI (kg/m2), median (IQR)	31.6 (8.5)
WC (cm), mean ± SD	109.8 ± 13.5
Marital status, N (%)	
Married	76 (76.0)
Divorced	4 (4.0)
Single	11 (11.0)
In a relationship	5 (5.0)
Widowed	4 (4.0)
Metabolic abnormality, N (%)	
Hypertension	67 (67.0)
Hyperglycemia	38 (38.0)
Hyperlipidemia	88 (88.0)
Medication, N (%)	
Antihypertensive treatment	31 (31.0)
Antidiabetic agents	26 (26.0)
Statins	28 (28.0)
Menopause, N (%) out of females	20 (67.2)
Lifestyle	
Smoking, N (%)	17 (17.0)
PAL (total MET-min/week), median (IQR)	792.0 (1827.0)
CESD-R, median (IQR)	14.0 (14.5)
RSES mean ± SD	31.2 ± 4.2
PCS-12, median (IQR)	47.4 (15.0)
MCS-12, median (IQR)	50.4 (15.0)
Biochemical parameters	
Glucose (mg/dL), median (IQR)	93.0 (16.0)
ΤC (mg/dL), mean ± SD	194.0 (36.9)
TG (mg/dL), median (IQR)	125.0 (80.0)
HDL (mg/dL), mean ± SD	49.6 (10.8)
LDL (mg/dL), mean ± SD	123.1 ± 35.7
SGOT (iu/L), median (IQR)	17.0 (7.0)
SGPT (iu/L), median (IQR)	17.0 (13.5)
γ-GT (iu/L), median (IQR)	18.0 (11.8)
ALP (U/L), mean ± SD	66.6 ± 17.5

The continuous variables are presented as mean±SD or median (IQR), according to their distribution, and the qualitative variables as counts (%). BMI: body mass index; WC: waist circumference; PAL: physical activity level as assessed by the International Physical Activity Questionnaire Short Form; CESD-R: Center for Epidemiologic Studies Depression Scale Revised; RSES: Rosenberg Self-Esteem Scale; PCS-12: Physical Component Score; MCS-12: Mental Composite Score; TC: total cholesterol; TG: triglycerides; HDL: high-density lipoprotein; LDL: low-density lipoprotein; SGOT: serum glutamic-oxaloacetic transaminase; SGPT: serum glutamic-pyruvic transaminase; γ-GT: γ-glutamyl transferase; ALP: alkaline phosphatase.

**Table 2:** Micronutrient intakes in participants.

Micronutrients	Daily Intakes Median (IQR)
Vitamin A (µg)	272.0 (231.9)
Beta-Carotene (µg)	901.4 (1228.8)
Alpha-Carotene (µg)	87.0 (294.4)
Lutein (+Zeaxanthin) (µg)	715.3 (1032.7)
Beta-Cryptoxanthin (µg)	20.1 (127.5)
Lycopene (µg)	1368.5 (2557.9)
Vitamin C (mg)	49.6 (55.0)
Vitamin D (µg)	1.9 (2.4)
Vitamin E (mg)	6.3 (4.4)
Thiamin (mg)	1.0 (0.5)
Riboflavin (mg)	1.2 (0.8)
Niacin (mg)	13.8 (9.8)
Pyridoxine (mg)	1.3 (0.8)
Folate Total (µg)	232.2 (192.6)
Cobalamin (µg)	2.3 (1.3)
Biotin (µg)	10.2 (9.7)
Pantothenic Acid (mg) *	3.4 ± 1.3
Choline (mg)	166.9 (122.6)
Vitamin K (µg)	53.8 (53.3)
Calcium (mg)*	707.5 ± 291.8
Iron (mg)	10.1 (6.0)
Sodium (mg)	1196.2 (785.5)
Potassium (mg)	1803.3 (872.1)
Phosphorus (mg)	876.4 (398.0)
Magnesium (mg)	200.4 (101.8)
Zinc (mg)	7.3 (3.2)
Copper (mg)	0.8 (0.4)
Manganese (mg)	1.9 (1.1)
Selenium (µg) *	78.7 ± 35.1
Chromium (mg)	0.03 (0.03)
Molybdenum (µg)	13.9 (27.4)

* All variables are presented as medians (IQR), with the exception of calcium, pantothenic acid, and selenium, which were normally distributed and are presented as mean ± standard deviation.

**Table 3:** The correlations between mental and physical health parameters and the dietary intake of micronutrients.

	CESD-R		RSES		PCS-12		MCS-12	
Micronutrients	Rho	*p*	Rho	*p*	Rho	*p*	Rho	*p*
Vitamin A (µg)	−0.072	0.554	0.134	0.275	−0.227	0.061	0.249	**0.038**
Beta-Carotene (µg)	−0.038	0.757	0.090	0.467	−0.161	0.161	0.144	0.235
Alpha-Carotene (µg)	−0.073	0.552	0.226	0.064	−0.042	0.734	0.149	0.220
Lutein (+Zeaxanthin) (µg)	0.013	0.916	0.170	0.166	−0.064	0.599	0.134	0.268
Beta-Cryptoxanthin (µg)	0.044	0.720	0.174	0.155	−0.087	0.475	0.110	0.367
Lycopene (µg)	−0.017	0.888	0.128	0.300	−0.019	0.877	0.044	0.719
Vitamin C (mg)	0.008	0.946	0.126	0.308	−0.026	0.834	0.293	**0.014**
Vitamin D (µg)	−0.046	0.708	−0.007	0.957	−0.127	0.297	0.166	0.170
Vitamin E (mg)	−0.029	0.813	0.090	0.463	0.012	0.923	0.222	0.063
Thiamin (mg)	0.201	0.096	−0.104	0.395	−0.081	0.503	0.169	0.160
Riboflavin (mg)	0.018	0.879	0.031	0.801	−0.085	0.483	0.264	**0.026**
Niacin (mg)	0.189	0.118	0.035	0.778	0.042	0.731	0.132	0.273
Pyridoxine (mg)	0.116	0.341	−0.013	0.914	−0.148	0.220	0.105	0.386
Folate Total (µg)	0.174	0.150	0.045	0.711	−0.063	0.605	0.238	**0.046**
Cobalamin (µg)	−0.020	0.871	0.067	0.586	−0.108	0.383	0.228	0.060
Biotin (µg)	0.017	0.892	−0.011	0.930	0.015	0.901	−0.048	0.692
Pantothenic Acid (mg)	0.057	0.642	−0.056	0.647	0.089	0.464	0.129	0.285
Choline (mg)	0.000	0.998	0.053	0.667	0.033	0.784	0.222	0.063
Vitamin K (µg)	0.000	0.998	0.050	0.688	−0.045	0.716	0.063	0.606
Calcium (mg)	0.055	0.649	−0.067	0.585	−0.057	0.639	0.133	0.269
Iron (mg)	0.103	0.398	0.024	0.845	−0.080	0.508	0.157	0.192
Sodium (mg)	−0.059	0.626	0.269	**0.026**	0.109	0.369	0.226	0.058
Potassium (mg)	0.151	0.211	−0.089	0.468	−0.030	0.808	0.174	0.146
Phosphorus (mg)	0.107	0.380	0.003	0.980	0.091	0.455	0.126	0.293
Magnesium (mg)	0.086	0.481	0.034	0.782	0.127	0.295	0.078	0.519
Zinc (mg)	−0.034	0.781	−0.031	0.799	−0.018	0.883	0.116	0.333
Copper (mg)	0.096	0.430	0.037	0.762	−0.004	0.971	0.141	0.244
Manganese (mg)	−0.100	0.408	0.175	0.151	0.104	0.393	0.190	0.113
Selenium (µg)	0.049	0.688	0.007	0.953	0.084	0.488	0.129	0.282
Chromium (mg)	0.313	0.009	−0.167	0.177	−0.068	0.581	−0.086	0.477
Molybdenum (µg)	0.073	0.549	−0.089	0.471	0.195	0.109	−0.018	0.879

CESD-R: Center for Epidemiologic Studies Depression Scale Revised; RSES: Rosenberg Self-Esteem Scale; PCS-12: Physical Component Score; MCS-12: Mental Composite Score. The correlation analysis was performed with Spearman’s rank correlation. The level of significance was set to 0.05. The significant *p* values are in bold.

**Table 4:** Linear regression models of mental health indices in association with dietary intake.

	Model 1 ^a^		Model 2 ^b^		Model 3 ^c^	
	Beta ± SE	*p*	Beta ± SE	*p*	Beta ± SE	*p*
Log CESD-R						
Chromium (mg)	2.258 ± 1.398	0.111	2.375 ± 1.407	0.097	2.559 ± 1.445	0.082
RSES						
Sodium (mg)	0.002 ± 0.001	0.055	0.001 ± 0.001	0.229	0.001 ± 0.001	0.146
Log MCS-12						
Vitamin A (µg)	3.047 × 10^−5^ ± 0.000	0.084	2.416 × 10^−5^ ± 0.000	0.165	2.314 × 10^−5^ ± 0.000	0.198
Vitamin C (mg)	0.000 ± 0.000	0.187	0.000 ± 0.000	0.585	9.210 × 10^−5^ ± 0.000	0.694
Riboflavin (mg)	0.043 ± 0.022	**0.050**	0.044 ± 0.021	**0.041**	0.047 ± 0.023	**0.044**
Folate Total (µg)	0.000 ± 0.000	0.146	0.000 ± 0.000	0.074	0.000 ± 0.000	0.114

CESD-R: Center for Epidemiologic Studies Depression Scale Revised; RSES: Rosenberg Self-Esteem Scale; MCS-12: Mental Composite Score. ^a^ crude model; ^b^ adjusted for age, sex, BMI; ^c^ adjusted for age, sex, BMI, physical activity level, smoking, medication (antihypertensive, antidiabetic, or statins). The values resulted from linear regression models. The level of significance was set to 0.05. The significant *p* results are in bold.

## Data Availability

Data will be made available upon the request ofthe corresponding authors.

## Supplementary Materials

The following supporting information can be downloaded at: https://www.mdpi.com/article/10.3390/nu15204464/s1. Table S1: Micronutrient intakes in males and females along with their respective Recommended Dietary Allowances (RDAs) and Adequate Intakes (AIs).
