# Supplementary material for: Mental Health Component Scale Is Positively Associated with Riboflavin Intake in People with Central Obesity
Source: Nutrients. 2023 Oct 21;15(20):4464. doi: 10.3390/nu15204464 (PMC10609858; doi:10.3390/nu15204464)
Supplement: Supplementary file 1 [file nutrients-15-04464-s001.zip › nutrients-2668566-supplementary.pdf]

Supplementary material related to:

Riboflavin intake is positively associated with the mental health component scale in people with central obesity

Charalampia Amerikanou[1], Aristea Gioxari[2], Stamatia-Angeliki Kleftaki[1], Evdokia Valsamidou[1], Antonia Zeaki[1], Andriana C. Kaliora[1]

[1]Department of Nutrition and Dietetics, School of Health Science and Education, Harokopio University of Athens, 70 El. Venizelou Ave., 17676 Athens, Greece

[2]Department of Nutritional Science and Dietetics, School of Health Science, University of the Peloponnese, Antikalamos, 24100 Kalamata-Messinia, Greece;

*Correspondence: A. C. Kaliora (akaliora@hua.gr), Department of Nutrition and Dietetics, School of Health Science and Education, Harokopio University of Athens, 70 El. Venizelou Ave., 17676 Athens, Greece, +30 2109549226

Supplementary Table S1. Micronutrient intakes in males and females along with the respective Recommended Dietary Allowances (RDAs) and Adequate Intakes (AIs)

Micronutrients	Males (N=39) Median (IQR) or Mean ± standard deviation	Adult Males Recommended Intakes	Females (N=61) Median (IQR) or Mean ± standard deviation	Adult Females Recommended Intakes
Vitamin A (μg/d)	267.0 (222.2)	900	297.6 (295.6)	700
Beta-Carotene (μg/d)	922.2 (968.7)	N/A	755.9 (1635.0)	N/A
Alpha-Carotene (μg/d)	87.6 (151.6)	N/A	67.2 (457.1)	N/A
Lutein (+ Zeaxanthin) (μg/d)	583.5 (1231.4)	N/A	794.6 (977.0)	N/A
Beta-Cryptoxanthin (μg/d)	16.0 (142.1)	N/A	26.6 (111.5)	N/A
Lycopene (μg/d)	1582.4 (2474.6)	N/A	1292.7 (2959.4)	N/A
Vitamin C (mg/d)	65.1 ± 40.7	90	46.3 (48.1)	75
Vitamin D (μg/d)	2.0 ± 1.7	10*	2.2 ± 1.6	10*
Vitamin E (mg/d)	6.2 (3.4)	15	7.1 ± 3.1	15
Thiamin (mg/d)	1.1 ± 0.5	1.2	1.1 ± 0.4	1.1
Riboflavin (mg/d)	1.2 (0.7)	1.3	1.2 (0.8)	1.1
Niacin (mg/d)	16.3 ± 8.4	16	13.3 (9.2)	14
Pyridoxine (mg/d)	1.5 ± 0.6	1.6	1.3 (0.8)	1.3
Folate Total (μg/d)	256.4 ± 117.3	400	234.4 (205.5)	400
Cobalamin (μg/d)	2.2 (1.5)	2.4	2.3 (1.2)	2.4
Biotin (μg/d)	12.9 ± 9.0	30*	9.5 (11.3)	30*
Pantothenic Acid (mg/d)**	2.9 ± 1.6	5*	3.4 ± 1.3	5*
Choline (mg/d)	171.5 (86.6)	550*	160.9 (145.3)	425*
Vitamin K (μg/d)	65.3 (41.2)	120*	53.1 (48.7)	90*
Calcium (mg/d)**	629.8 (273.7)	1000*	710.3 ± 285.2	1000*
Iron (mg/d)	10.2 (4.1)	8	9.7 (7.5)	8
Sodium (mg/d)	1307.7 (776.8)	1500*	1262.5 ±692.5	1500*
Potassium (mg/d)	1826.3 (1077.8)	3400	1941.6 ± 764.9	2600
Phosphorus (mg/d)	796.0 (316.2)	550	938.7 (316.4)	550
Magnesium (mg/d)	195.5 (146.3)	420	212.1 ± 76.2	320
Zinc (mg/d)	7.3 (3.4)	11	7.3 (3.2)	8
Copper (mg/d)	0.8 (0.4)	0.9	0.8 (0.4)	0.9
Manganese (mg/d)	1.8 (0.8)	2.3*	2.0 (1.2)	1.8*
Selenium (μg/d)**	80.1 ± 36.9	55	77.8 ± 34.1	55
Chromium (mg/d)	0.03 (0.03)	0.02 *	0.03 (0.03)	0.03*
Molybdenum (μg/d)	16.2 (36.5)	45	12.7 (25.6)	45

*Recommended Dietary Allowances (RDAs) in ordinary type and Adequate Intakes (AIs) are followed by an asterisk (*). RDAs and AIs may both be used as goals for individual intake. RDAs are set to meet the needs of almost all (97 to 98 percent) individuals in a

group. The AI is believed to cover the needs of all individuals in the group, but a lack of data or uncertainty in the data prevents being able to specify with confidence the percentage of individuals covered by this intake. [1,2]
[**]Variables are presented as median (IQR) or mean ± standard deviation depending on their distribution in each sex.

References

1. Institute of Medicine (2003) Dietary Reference Intakes: Guiding Principles for Nutrition Labeling and Fortification. Washington, DC: The National Academies Press. https://doi.org/10.17226/10872.

2. National Academies of Sciences, Engineering, and Medicine (2019) Dietary Reference Intakes for Sodium and Potassium. Washington, DC: The National Academies Press. https://doi.org/10.17226/25353.
